# Life Satisfaction, Interpersonal Relationships, and Learning Influence Withdrawal from School: A Study among Junior High School Students in Japan

**DOI:** 10.3390/ijerph15102309

**Published:** 2018-10-20

**Authors:** Sachiko Inoue, Tsuguhiko Kato, Takashi Yorifuji

**Affiliations:** 1 Department of Nursing Science, Faculty of Health and Welfare Science, Okayama Prefectural University, 111 Kuboki, Soja, Okayama 719-1197, Japan; 2 Department of Social Medicine, National Center for Child Health and Development, 2-10-1 Okura, Setagaya-ku, Tokyo 157-8535, Japan; kato-tg@ncchd.go.jp; 3 Department of Human Ecology, Okayama University Graduate School of Environmental and Life Science, 1-1-1Tsushimanaka, Kita-ku, Okayama 700-8350, Japan; yorichan@md.okayama-u.ac.jp

**Keywords:** absenteeism, adaptation, epidemiology, school adjustment, school withdrawal

## Abstract

School absenteeism, particularly among junior high school students, has increased annually in Japan. This study demonstrates the relationship between subjective adjustment to school life and students’ absenteeism. Data were collected from 17,378 junior high school students in Japan. A longitudinal design was used for the study. Teachers were asked to distribute the Adaptation Scale for School Environments on Six Spheres (ASSESS) questionnaire to junior high school students and ask the students to fill out the questionnaire at the beginning of the 2014 academic year in April 2014, and the relationship between their subjective adjustment and absenteeism as measured by the total number of absent days during the 2014 academic year was evaluated by logistic regression and a survival analysis model. Low life satisfaction was associated with absences. The corresponding odds ratio (OR) was higher for seventh graders (OR 3.29, confidence interval (CI): 2.41–4.48, hazard ratio (HR) 5.57, CI: 3.51–8.84) than for students in other grades. Interpersonal relationships were significantly related to absenteeism for seventh and eighth graders in the group with scores less than 39 points. Lower adjustment to learning seemed to be related to absenteeism for seventh and eighth graders. For effective interventions, a well-designed study that uses detailed information regarding life-related covariates is necessary.

## 1. Introduction 

Children spend a substantial amount of time at school. School provides an important community that has a range of influences on children, and school life affects children’s behavioral outcomes [1,2,3,4]. Individual behavior that is characterized in a community is called social capital [5]. Previous studies have revealed relationships between school social capital and child development and behavior. Students who do not have a sense of belonging to their school are more likely to exhibit violent behavior [6]. Moreover, school-related social capital prevents depression and burnout, and is associated with decreased suicide risk among female students [7]. Although the school environment influences children’s health and behavior, comprehensive evidence regarding these associations is currently lacking.

Several previous studies reported that a range of problems among children, including physical health, psychological health, social relationships, and living environment, are all related to difficulties at school, as measured by absenteeism [8,9,10,11,12,13,14,15]. Various lifestyle factors and school-related variables are reported to be associated with absenteeism. Meanwhile, empirical evidence regarding teachers’ practices suggests that children’s perceptions of adjustment to school with respect to teacher support, friendship, and non-intrusive relationships determine perceptions of individual behavior in the school community. However, because a small number of studies have evaluated the effects of adjustment to school life on children’s behavioral outcomes so far, the evidence of the impact of adjustment on children’s absenteeism remains scant [16,17,18]. Therefore, in the current study, we examined the relationship between students’ perception of adjustment to school and long-term absenteeism among junior high school students using an instrument with six perception-related items relating to students’ school life. Evaluating with this measurement of both individual factors and relational behavior variables in the school community can contribute to an understanding of school absenteeism among children.

## 2. Materials and Methods

### 2.1. Data Source

A longitudinal design with a one-year follow-up period was used in this study. Baseline questionnaires were distributed to students in the seventh to ninth grades at 38 junior high schools in Okayama Prefecture in western Japan at the beginning of the 2014 academic year. The baseline questionnaire was distributed to a total of 18,729 students. The teachers at each school collected the questionnaires from the students and sent the questionnaires to the researchers. As a result, 17,378 students responded to the questionnaire. Twelve months later, information on each student’s absences was collected from each school according to the record at the end of the 2014 academic year. Each student was assigned an identification number only for the purpose of this study. We linked the baseline data of the questionnaires and absenteeism data using Excel (Microsoft Office 2013, Microsoft, Tokyo, Japan).

### 2.2. Exposure Variable

Subjective Adjustment to School Life Scores on the Adaptation Scale for School Environments on Six Spheres (ASSESS), which measures children’s subjective adjustment to school life, were used as the exposure variable for this study [19,20]. ASSESS is introduced in junior high school to promote class and student development. Scores are measured on the six subscales of: life satisfaction, teacher support, friendship, social skills, non-intrusive relationships, and learning. Another subscale for interpersonal relationships can be created by combining the teacher support, friendship, social skills, and non-intrusive relationship subscales. Those four variables seem to compensate for each other. Thus, we used the comprehensive variable of interpersonal relationships. The two variables of life satisfaction and learning are not related to the variable of interpersonal relationships. Therefore, we used the three subscales of life satisfaction, learning, and interpersonal relationships in this study. For each item on ASSESS, response values ranged from 1 to 4, with 1 indicating lower adjustment and 4 indicating higher adjustment. Data were entered into a programmed Excel datasheet, and t-scores were calculated for the original six subscales and then recalculated for interpersonal relationship scores with the combined four subscales of teacher support, friendship, social skills, and non-intrusive relationships. The average t-score value was 50, which is a standardized score with standard deviation (SD) = 10. Thus, for each subscale, ASSESS scores were classified into three categories (less than 39, 40 to 49, and greater than 50). These categories were established by the developer of ASSESS, and are used by teachers who use ASSESS in their practice. We used the data of ASSESS collected at the beginning of the academic year in April 2014 and data on school absenteeism during the 2014 academic year which were obtained in March 2015. In Japan, the school year starts in April, and the students’ subjective adjustment in this period could be more influenced by class atmosphere which consists of new friends, new teachers, and a new classroom.

### 2.3. Outcome Variable: School Absenteeism

School absenteeism data were used as the outcome variable. Each school has a built-in reporting system for students who reach three days of absence from school. We used this information and linked it to the ASSESS data. Two classification approaches were used for the school absenteeism outcome variable. One approach involved two categories with a cutoff point of 10 days (less than 9 days or 10 days or more), and the other approach involved two categories with a cutoff point of 30 days (less than 29 days or 30 days or more). In the Japanese education system, a student is considered to have withdrawn from school when the total number of absent days reaches 30 days during an academic year. Thus, we used this definition for the outcome variable of school absenteeism. This defines only the event of having withdrawn from school and includes many reasons for being absent.

### 2.4. Data Analysis and Ethical Issues

Descriptive analysis of the study participants was performed. A logistic model was then used to evaluate the relationship between subjective adjustment to school life and school absenteeism. Subsequently, odds ratios (ORs) and 95% confidence intervals (CIs) were calculated. Using the logistic model, we first analyzed all data together and then assessed data separately for each grade (seventh, eighth, and ninth grades). An adjusted model was not applied due to insufficient information regarding covariates. To generate supplementary information, we used a Cox proportional hazard model to conduct survival analyses to evaluate the relationships between ASSESS subscale scores (which were treated as continuous variables) and the time of occurrence of school absenteeism (defined as the time (month) between April and March of the 2014 academic year when a student’s total absences from school reached more than 30 days). A hazard ratio (HR) was then estimated. All covariates in these analyses were adjusted for lack of information. We used SPSS version 23 (IBM, Japan, Tokyo) for all statistical analyses. This study was reviewed and approved by the Okayama Prefectural University Institutional Review Board on 25 November 2014 (No. 428).

## 3. Results

**Figure 1:**
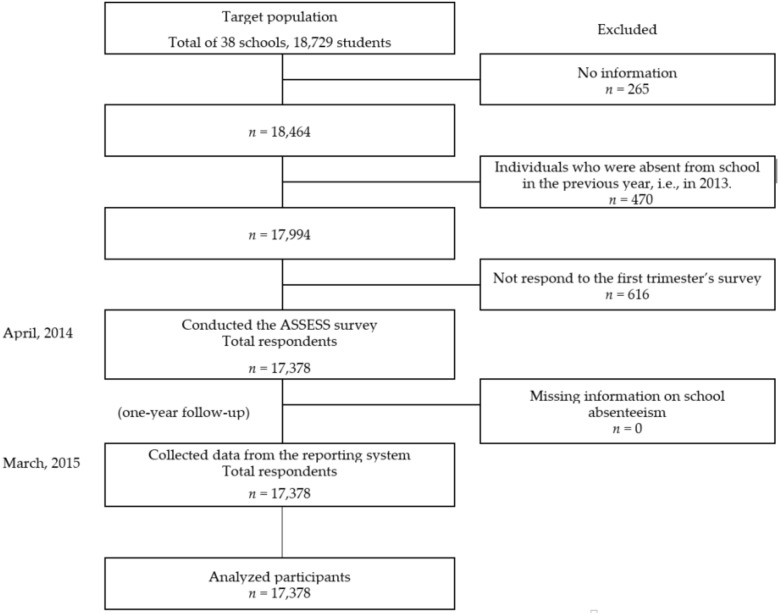
Selection of participants.

A total of 17,378 participants completed the questionnaires (Figure 1). Cronbach’s alpha was 0.793. The overall rate of school absenteeism was 1.49% (*n* = 258) among the current study participants. We analyzed data on the relationship between junior high school students’ subjective adjustment to school life and absenteeism in Okayama Prefecture, Japan. We used six subscales as a measure of subjective adjustment to school life, including life satisfaction, teacher support, friendship, social skills, non-intrusive relationships, and learning. Descriptive analysis results revealed that the proportion of students with more than 10 days absent increased up to 12.2% in life satisfaction, 9.4% in teacher support, 9.2% in friendship, and 8.0% in social skills in the group with scores of less than 39 points (more than 10 points lower than the mean value of 50) among the seventh graders. The proportions of those four variables were similarly increased among the eighth and ninth graders. This tendency was not shown in the lower score of subjective adjustment of non-intrusive relationship and learning (Table 1). We found that students’ subjective adjustment with respect to life satisfaction, adjustment to learning, and interpersonal relationships was related to absenteeism as indicated by associations between scores of less than 39 points on these subscales and more than 10 days or more than 30 days of absences (Table 2). Analysis by grade indicated that the association between low life satisfaction and absenteeism had a higher OR for seventh graders (OR 3.29, CI 2.41–4.48) than for students in other grades. In Table 3, survival analysis using a logistic regression model produced results similar to this finding (HR 5.57, CI 3.51–8.84). For the subscales of learning and interpersonal relationships, there were significant relationships between poor subjective adjustment and absenteeism in the overall analysis, with significant associations between scores of less than 39 points and absenteeism for seventh and eighth graders.

## 4. Discussion

This large epidemiological study of junior high school students in Japan evaluated the effect of subjective adjustment to school on absenteeism. This study found an overall relationship between students’ perception regarding adjustment to school life and school absenteeism.

### 4.1. Lower Life Satisfaction Affects School Absenteeism

For children with scores of 39 points or lower, a significant relationship was observed between overall subjective adjustment to school life and school absenteeism. In particular, low life satisfaction was associated with a particularly high risk of absenteeism among seventh graders. The seventh grade is a transitional stage from elementary school to junior high school. As part of this transition, children must simultaneously devote effort to many different tasks, such as commuting to a new school [21], relating to new classmates and teachers, and studying. Students’ difficulties tend to increase after they enter junior high school. Many children are unaware of their own problems and have difficulty determining what they think and feel. These situations may be identified by symptoms of fatigue, hypodynamia, and irritation [16]. Pressures associated with this situation may push students toward refusing to attend school.

Furthermore, it has been reported that separation anxiety is a fundamental factor in school withdrawal [22,23]. The parent–child relationship at home is also a critical factor in children’s school absenteeism, even when students are at junior high school [24]. Parental behaviors, such as autonomy support, involvement, and structure are reported to be additional important factors in children’s school adjustment [15]. Simultaneously, the consequences of school mobility are thought to affect children’s social adjustment [25]. Students who experience social mobility as they enter junior high school may require support to socially adjust to their community and neighbors. Community nurses may be able to play a role in this situation by collaborating with school teachers, potentially helping to increase children’s life satisfaction.

### 4.2. Adjustment to Learning and Absenteeism

We found an association between learning and absenteeism in an analysis of all grades and in separate analyses of the seventh and eighth grades. However, this association was not significant for children in the ninth grade. One previous study examined the relationship between cognitive function and the prevalence of fatigue [26]. The findings revealed that fatigue among junior high school students was associated with reduced cognitive function. Furthermore, sleep disturbance is also reported to be related to academic performance [27]. School withdrawal related to symptoms of fatigue and sleep disturbance may be attributed to a failure of learning adjustment.

Annual statistics in Japan indicate that approximately 10% of absenteeism among junior high school students can be attributed to academic failure [28], while nearly 5% of students exhibit learning difficulties [29]. Because only encouraging high academic performance may increase children’s anxiety, screening and appropriate consultations in fields ranging from education to medicine may provide a more effective approach. Further studies involving multidisciplinary collaborations that examine students’ difficulties in adjusting to learning are necessary.

### 4.3. Interpersonal Relationships and Absenteeism

Regarding the interpersonal relationship subscale, which incorporates the four subscales of teacher support, friendship, social skills, and non-intrusive relationships, lower scores in the 40–49 group were related to school absenteeism. Students are reported to become more sensitive to interpersonal relationships over time, and difficulties in relationships may be a major cause of avoiding school and exhibiting further social withdrawal [30,31]. In addition, school withdrawal at the period of junior high school has been reported to be related to communication skills in school settings among the student group in the transitional phase of adjustment to junior high school, and school absenteeism has been attributed to school-life skills, including communication skills [32]. Moreover, aggression toward peers has been reported as a potential predictor of school withdrawal [10]. However, the current study revealed no significant relationship among children with scores below 39 points. One possible explanation for this finding is that individuals who experienced poor subjective adjustment to school life, particularly with respect to interpersonal relationships, already exhibited absenteeism. Thus, in the current analysis, the true association between interpersonal relationships and absenteeism may have been obscured.

### 4.4. Limitations

ASSESS and absenteeism data were collected from 38 schools in one municipal city in Okayama Prefecture. More than 90% of junior high school students in the municipality responded to the questionnaire; however, students who were already exhibiting absenteeism might have missed the opportunity to respond to the questionnaire. This issue may have introduced selection bias. In addition, we could not adjust for gender and other sociodemographic variables in the statistical model. Therefore, the effects of possible confounders cannot be excluded. Furthermore, there is little information regarding the factors included in children’s feelings of life satisfaction (the life satisfaction subscale). A well-designed study that includes rich, wide-ranging information regarding exposure variables and possible confounders is necessary.

## 5. Conclusions

Our study revealed that students’ subjective adjustment to school life affects their school absenteeism. Interpersonal relationships and overall life satisfaction may play important roles for students during puberty. Therefore, it is necessary to conduct more detail-oriented studies to construct effective interventions.

## Figures and Tables

**Table 1:** Descriptive data on study participants.

Gender Informationand ASSESS Scores		Seventh Grade					Eighth Grade					Ninth Grade				
		Total	Absent More Than				Total	Absent More Than				Total	Absent More Than			
			10 Days		30 Days			10 Days		30 Days			10 Days		30 Days	
			*n*	%	*n*	%		*n*	%	*n*	%		*n*	%	*n*	%
Gender																
	Boy	2623	110	(4.2)	40	(1.5)	2366	128	(5.4)	44	(1.9)	2686	90	(3.4)	35	(1.3)
	Girl	2404	142	(5.9)	54	(2.2)	2239	137	(6.1)	51	(2.3)	2469	91	(3.7)	35	(1.4)
	Missing	1179	75	(6.4)	25	(2.1)	1352	70	(5.2)	28	(2.1)	626	28	(4.5)	14	(2.2)
Life satisfaction																
	More than 50	4079	165	(4.0)	45	(1.1)	3744	156	(4.2)	42	(1.1)	4124	108	(2.6)	35	(0.8)
	40–49	1473	77	(5.2)	27	(1.8)	1418	83	(5.9)	34	(2.4)	978	38	(3.9)	15	(1.5)
	Less than 39	501	61	(12.2)	30	(6.0)	605	58	(9.6)	21	(3.5)	455	26	(5.7)	9	(2.0)
Teacher support																
	More than 50	3952	171	(4.3)	56	(1.4)	3682	179	(4.9)	55	(1.5)	4165	124	(3.0)	41	(1.0)
	40–49	1503	76	(5.1)	21	(1.4)	1539	73	(4.7)	29	(1.9)	1021	28	(2.7)	9	(0.9)
	Less than 39	596	56	(9.4)	25	(4.2)	540	45	(8.3)	13	(2.4)	372	20	(5.4)	9	(2.4)
Friendship																
	More than 50	4122	189	(4.6)	52	(1.3)	3523	160	(4.5)	51	(1.4)	3750	106	(2.8)	30	(0.8)
	40–49	1436	68	(4.7)	30	(2.1)	1551	89	(5.7)	28	(1.8)	1346	43	(3.2)	17	(1.3)
	Less than 39	498	46	(9.2)	20	(4.0)	692	48	(6.9)	18	(2.6)	460	23	(5.0)	12	(2.6)
Social skills																
	More than 50	3533	156	(4.4)	41	(1.2)	3405	153	(4.5)	46	(1.4)	3396	97	(2.9)	26	(0.8)
	40–49	1758	86	(4.9)	30	(1.7)	1753	92	(5.2)	37	(2.1)	1439	42	(2.9)	20	(1.4)
	Less than 39	765	61	(8.0)	31	(4.1)	609	52	(8.5)	14	(2.3)	724	33	(4.6)	13	(1.8)
Non-intrusive relationship																
	More than 50	3814	193	(5.1)	66	(1.7)	3291	145	(4.4)	43	(1.3)	3049	87	(2.9)	29	(1.0)
	40–49	1734	80	(4.6)	26	(1.5)	1865	102	(5.5)	32	(1.7)	1958	59	(3.0)	17	(0.9)
	Less than 39	507	30	(5.9)	10	(2.0)	606	50	(8.3)	22	(3.6)	552	26	(4.7)	13	(2.4)
Learning																
	More than 50	3953	161	(4.1)	56	(1.4)	3415	142	(4.2)	44	(1.3)	2755	74	(2.7)	27	(1.0)
	40–49	1444	87	(6.0)	27	(1.9)	1496	76	(5.1)	27	(1.8)	1804	59	(3.3)	21	(1.2)
	Less than 39	658	55	(8.4)	19	(2.9)	854	79	(9.3)	26	(3.0)	999	39	(3.9)	11	(1.1)
Total		6055	303	(5.0)	102	(1.7)	5765	297	(5.2)	97	(1.7)	5558	172	(3.1)	59	(1.1)

**Table 2:** Odds ratios and confidence intervals for the association between first trimester Adaptation Scale for School Environments on Six Spheres (ASSESS) scores and total days absent (over 10 or 30 days).

ASSESS Scores	All Grades				Seventh Grade				Eighth Grade				Ninth Grade			
	Absent More Than				Absent More Than				Absent More Than				Absent More Than			
	10 Days		30 Days		10 Days		30 Days		10 Days		30 Days		10 Days		30 Days	
	OR	95%CI	OR	95%CI	OR	95%CI	OR	95%CI	OR	95%CI	OR	95%CI	OR	95%CI	OR	95%CI
Life satisfaction																
More than 50	Reference		Reference		Reference		Reference		Reference		Reference		Reference		Reference	
40–49	1.40	(1.23, 1.60)	1.45	(1.22, 1.72)	1.30	(1.06, 1.60)	1.31	(0.99, 1.73)	1.31	(1.06, 1.62)	1.43	(1.09, 1.88)	1.58	(1.20, 2.06)	1.50	(1.03, 2.19)
Less than 39	2.06	(1.75, 2.42)	2.73	(2.25, 3.32)	2.30	(1.77, 3.00)	3.29	(2.41, 4.48)	1.83	(1.41, 2.38)	2.44	(1.78, 3.34)	2.00	(1.42, 2.80)	2.25	(1.45, 3.50)
Learning																
More than 50	Reference		Reference		Reference		Reference		Reference		Reference		Reference		Reference	
40–49	1.21	(1.07, 1.38)	1.27	(1.07, 1.50)	1.25	(1.02, 1.55)	1.51	(1.15, 1.97)	1.35	(1.09, 1.68)	1.23	(0.93, 1.64)	1.19	(0.93, 1.54)	1.22	(0.87, 1.73)
Less than 39	1.76	(1.53, 2.03)	1.92	(1.60, 2.31)	1.86	(1.44, 2.39)	2.15	(1.56, 2.95)	2.14	(1.70, 2.69)	2.35	(1.77, 3.13)	1.53	(1.16, 2.04)	1.47	(0.99, 2.18)
Interpersonal relationship																
More than 50	Reference		Reference		Reference		Reference		Reference		Reference		Reference		Reference	
40–49	1.32	(1.17, 1.48)	1.46	(1.25, 1.71)	1.24	(1.02, 1.52)	1.48	(1.15, 1.90)	1.36	(1.12, 1.66)	1.63	(1.27, 2.09)	1.34	(1.05, 1.71)	1.16	(0.83, 1.63)
Less than 39	1.79	(1.32, 2.41)	1.79	(1.20, 2.65)	1.53	(0.90, 2.62)	1.58	(0.79, 3.16)	1.84	(1.16, 2.94)	1.84	(1.00, 3.39)	2.00	(1.11, 3.61)	1.95	(0.89, 4.27)

OR: odds ratio, CI: confidence interval. Crude odds ratios were calculated in those analyses any variables were adjusted for.

**Table 3:** Hazard ratios and confidence intervals for the association between first trimester ASSESS scores and total days absent (over 10 or 30 days).

ASSESS Scores	All Grades				Seventh Grade				Eighth Grade				Ninth Grade			
	Absent More Than				Absent More Than				Absent More Than				Absent More Than			
	10 Days		30 Days		10 Days		30 Days		10 Days		30 Days		10 Days		30 Days	
	HR	95%CI	HR	95%CI	HR	95%CI	HR	95%CI	HR	95%CI	HR	95%CI	HR	95%CI	HR	95%CI
Life satisfaction																
More than 50	Reference		Reference		Reference		Reference		Reference		Reference		Reference		Reference	
40–49	1.44	(1.22, 1.70)	1.95	(1.47, 2.60)	1.30	(0.99, 1.70)	1.66	(1.03, 2.68)	1.42	(1.09, 1.86)	2.15	(1.37, 3.38)	1.49	(1.03, 2.16)	1.81	(0.99, 3.32)
Less than 39	2.66	(2.20, 3.21)	3.80	(2.79, 5.17)	3.17	(2.37, 4.26)	5.57	(3.51, 8.84)	2.37	(1.75, 3.20)	3.12	(1.85, 5.27)	2.23	(1.45, 3.42)	2.36	(1.13, 4.90)
Learning																
More than 50	Reference		Reference		Reference		Reference		Reference		Reference		Reference		Reference	
40–49	1.26	(1.07, 1.49)	1.26	(0.95, 1.67)	1.49	(1.15, 1.94)	1.32	(0.84, 2.09)	1.22	(0.93, 1.62)	1.40	(0.87, 2.27)	1.22	(0.87, 1.72)	1.19	(0.67, 2.10)
Less than 39	1.89	(1.58, 2.26)	1.81	(1.33, 2.48)	2.10	(1.54, 2.85)	2.05	(1.22, 3.45)	2.28	(1.73, 3.00)	2.38	(1.46, 3.86)	1.46	(0.99, 2.15)	1.12	(0.56, 2.26)
Interpersonal relationship																
More than 50	Reference		Reference		Reference		Reference		Reference		Reference		Reference		Reference	
40–49	1.45	(1.24, 1.69)	1.86	(1.44, 2.40)	1.47	(1.15, 1.88)	2.08	(1.38, 3.14)	1.61	(1.26, 2.04)	1.88	(1.25, 2.84)	1.16	(0.83, 1.61)	1.51	(0.87, 2.62)
Less than 39	1.76	(1.20, 2.58)	2.06	(1.08, 3.91)	1.56	(0.80, 3.04)	2.41	(0.87, 6.66)	1.80	(1.00, 3.24)	1.42	(0.44, 4.54)	1.94	(0.90, 4.15)	2.68	(0.82, 8.70)

HR: hazard ratio, CI: confidence interval. Crude hazard ratios were calculated in those analyses and any variables were adjusted for.
